# Aqua­(4,4′-dimethyl-2,2′-bipyridine-κ^2^ *N*,*N*′)(nitrato-κ*O*)(nitrato-κ^2^ *O*,*O*′)zinc

**DOI:** 10.1107/S1600536811050227

**Published:** 2011-11-30

**Authors:** Sadif A. Shirvan, Sara Haydari Dezfuli

**Affiliations:** a Department of Chemistry, Omidieh Branch, Islamic Azad University, Omidieh, Iran

## Abstract

In the title compound, [Zn(NO_3_)_2_(C_12_H_12_N_2_)(H_2_O)], the Zn^II^ atom is six-coordinated in a distorted octa­hedral geometry by two N atoms from a chelating 4,4′-dimethyl-2,2′-bipyridine ligand, one water O atom, one O atom from a monodentate nitrate anion and two O atoms from a chelating nitrate anion. In the crystal, there are aromatic π–π contacts between the pyridine rings [centroid–centroid distances = 3.9577 (13) Å] and inter­molecular O—H⋯O and C—H⋯O hydrogen bonds.

## Related literature

For related structures, see: Ahmadi *et al.* (2008 ▶); Alizadeh *et al.* (2010 ▶); Amani *et al.* (2009 ▶); Bellusci *et al.* (2008 ▶); Hojjat Kashani *et al.* (2008 ▶); Kalateh *et al.* (2008 ▶, 2010 ▶); Sakamoto *et al.* (2004 ▶); Sofetis *et al.* (2006 ▶); Willett *et al.* (2001 ▶); Yoshikawa *et al.* (2003 ▶); Yousefi *et al.* (2008 ▶).
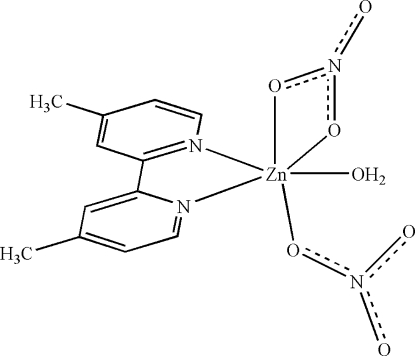


## Experimental

### 

#### Crystal data

[Zn(NO_3_)_2_(C_12_H_12_N_2_)(H_2_O)]
*M* *_r_* = 391.66
Monoclinic, 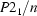

*a* = 10.9266 (5) Å
*b* = 8.5717 (3) Å
*c* = 16.8073 (7) Å
β = 97.873 (4)°
*V* = 1559.33 (11) Å^3^
*Z* = 4
Mo *K*α radiation
μ = 1.62 mm^−1^
*T* = 120 K
0.5 × 0.4 × 0.31 mm

#### Data collection

Bruker APEXII CCD area-detector diffractometer
Absorption correction: multi-scan (*SADABS*; Bruker, 2001 ▶) *T*_min_ = 0.460, *T*_max_ = 0.596
16739 measured reflections
4193 independent reflections
3596 reflections with *I* > 2σ(*I*)
*R*_int_ = 0.054

#### Refinement

*R*[*F* ^2^ > 2σ(*F* ^2^)] = 0.037
*wR*(*F* ^2^) = 0.084
*S* = 1.08
4193 reflections
227 parameters
H atoms treated by a mixture of independent and constrained refinement
Δρ_max_ = 0.50 e Å^−3^
Δρ_min_ = −0.65 e Å^−3^

### 

Data collection: *APEX2* (Bruker, 2007 ▶); cell refinement: *SAINT* (Bruker, 2007 ▶); data reduction: *SAINT*; program(s) used to solve structure: *SHELXS97* (Sheldrick, 2008 ▶); program(s) used to refine structure: *SHELXL97* (Sheldrick, 2008 ▶); molecular graphics: *SHELXTL* (Sheldrick, 2008 ▶); software used to prepare material for publication: *SHELXTL*.

## Supplementary Material

Crystal structure: contains datablock(s) global, I. DOI: 10.1107/S1600536811050227/bt5709sup1.cif

Structure factors: contains datablock(s) I. DOI: 10.1107/S1600536811050227/bt5709Isup2.hkl

Additional supplementary materials:  crystallographic information; 3D view; checkCIF report

## Figures and Tables

**Table 1:** Selected bond lengths (Å)

Zn1—N1	2.1157 (16)
Zn1—N2	2.0727 (17)
Zn1—O1	2.0965 (15)
Zn1—O2	2.5143 (16)
Zn1—O4	2.0340 (15)
Zn1—O7	2.0752 (15)

**Table 2:** Hydrogen-bond geometry (Å, °)

*D*—H⋯*A*	*D*—H	H⋯*A*	*D*⋯*A*	*D*—H⋯*A*
O7—H7*A*⋯O4^i^	0.85 (3)	1.90 (3)	2.724 (2)	163 (3)
O7—H7*B*⋯O3^ii^	0.87 (4)	1.94 (4)	2.799 (2)	174 (3)
C5—H5⋯O2^iii^	0.93	2.53	3.420 (2)	161
C8—H8⋯O2^iii^	0.93	2.36	3.230 (2)	156
C11—H11⋯O6^iv^	0.93	2.44	3.284 (3)	151

Symmetry codes: (i) 
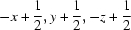
; (ii) 
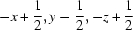
; (iii) 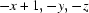; (iv) 
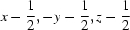
.

**Fig. 1.**
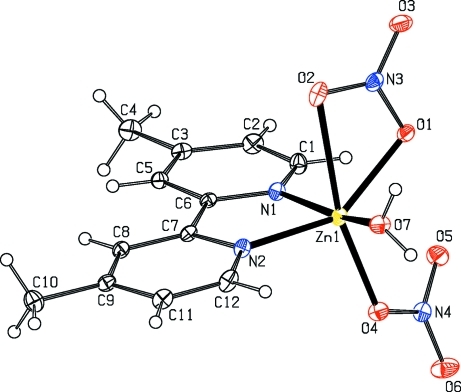
The molecular structure of the title molecule, with the atom-numbering scheme. Displacement ellipsoids are drawn at the 30% probability level.

**Fig. 2.**
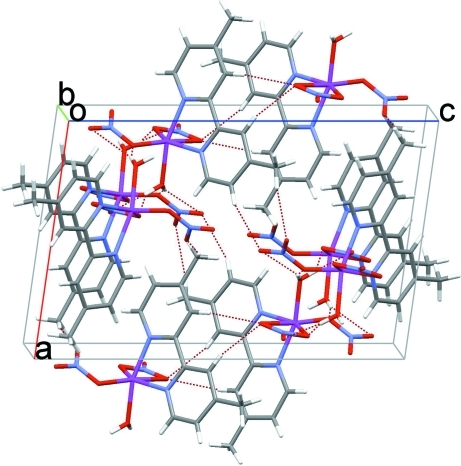
Unit-cell packing diagram for title compound.

## supplementary crystallographic information

### Comment

4,4'-Dimethyl-2,2'-bipyridine (4,4'-dmbipy), is a good bidentate ligand, and numerous complexes with 4,4'-dmbipy have been prepared, such as that of mercury (Kalateh *et al.*, 2008; Yousefi *et al.*, 2008), indium (Ahmadi *et al.*, 2008), iron (Amani *et al.*, 2009), platin (Hojjat Kashani *et al.*, 2008), manganese (Sakamoto *et al.*, 2004), silver (Bellusci *et al.*, 2008), gallium (Sofetis *et al.*, 2006), copper (Willett *et al.*, 2001), iridium (Yoshikawa *et al.*, 2003), cadmium (Kalateh *et al.*, 2010) and zinc (Alizadeh *et al.*, 2010). Here, we report the synthesis and structure of the title compound.

In the molecule of the title compound, (Fig. 1), the Zn^II^ atom is six-coordinated in a distorted octahedral configurations by two N atoms from the chelating 4,4'-dimethyl-2,2'-bipyridine, one O atom from water, one O atom from mono dentate nitrate anion and two O atoms from the chelating nitrate anion. The Zn—O and Zn—N bond lengths and angles are collected in Table 1.

In the crystal structure, intermolecular O—H···O and C—H···O hydrogen bonds (Table 2 & Fig. 2) link the molecules, in which they may be effective in the stabilization of the structure. There are also aromatic π-π contacts between the pyridine rings, *Cg*3···*Cg*4^i^ [symmetry cods: (i) 1-*X*,-Y,-*Z*, where *Cg*3 and *Cg*4 are centroids of the rings (N1/C1—C3/C5—C6) and (N2/C7—C9/C11—C12), respectively] further stabilize the structure, with centroid-centroid distances of 3.9577 (13) Å.

### Experimental

For the preparation of the title compound, a solution of 4,4'-dimethyl-2,2'-bipyridine (0.20 g, 1.10 mmol) in methanol (10 ml) was added to a solution of Zn(NO_3_)_2_.6H_2_O (0.33 g, 1.10 mmol) in acetonitrile (10 ml) and the resulting colorless solution was stirred for 20 min at 313 K. This solution was left to evaporate slowly at room temperature. After one week, colorless prismatic crystals of the title compound were isolated (yield 0.33 g, 76.6%).

### Refinement

H atoms bonded to C were positioned geometrically, with C—H=0.93Å for aromatics H and constrained to ride on their parent atoms, with *U*_iso_(H)=1.2*U*_eq_. H atoms bonded to O were freely refined.

### Crystal data

**Table:**

[Zn(NO_3_)_2_(C_12_H_12_N_2_)(H_2_O)]	*F*(000) = 800
*M**_r_* = 391.66	*D*_x_ = 1.668 Mg m^−^^3^
Monoclinic, *P*2_1_/*n*	Mo *K*α radiation, λ = 0.71073 Å
Hall symbol: -P 2yn	Cell parameters from 16739 reflections
*a* = 10.9266 (5) Å	θ = 2.1–29.2°
*b* = 8.5717 (3) Å	µ = 1.62 mm^−^^1^
*c* = 16.8073 (7) Å	*T* = 120 K
β = 97.873 (4)°	Prism, colorless
*V* = 1559.33 (11) Å^3^	0.5 × 0.4 × 0.31 mm
*Z* = 4	

### Data collection

**Table:**

Bruker APEXII CCD area-detector diffractometer	3596 reflections with *I* > 2σ(*I*)
graphite	*R*_int_ = 0.054
ω scans	θ_max_ = 29.2°, θ_min_ = 2.1°
Absorption correction: multi-scan (*SADABS*; Bruker, 2001)	*h* = −14→14
*T*_min_ = 0.460, *T*_max_ = 0.596	*k* = −11→11
16739 measured reflections	*l* = −20→23
4193 independent reflections	

### Refinement

**Table:**

Refinement on *F*^2^	Primary atom site location: structure-invariant direct methods
Least-squares matrix: full	Secondary atom site location: difference Fourier map
*R*[*F*^2^ > 2σ(*F*^2^)] = 0.037	Hydrogen site location: inferred from neighbouring sites
*wR*(*F*^2^) = 0.084	H atoms treated by a mixture of independent and constrained refinement
*S* = 1.08	*w* = 1/[σ^2^(*F*_o_^2^) + (0.0418*P*)^2^ + 0.5925*P*] where *P* = (*F*_o_^2^ + 2*F*_c_^2^)/3
4193 reflections	(Δ/σ)_max_ = 0.011
227 parameters	Δρ_max_ = 0.50 e Å^−^^3^
0 restraints	Δρ_min_ = −0.65 e Å^−^^3^

### Special details

**Table:**

Geometry. All e.s.d.'s (except the e.s.d. in the dihedral angle between two l.s. planes) are estimated using the full covariance matrix. The cell e.s.d.'s are taken into account individually in the estimation of e.s.d.'s in distances, angles and torsion angles; correlations between e.s.d.'s in cell parameters are only used when they are defined by crystal symmetry. An approximate (isotropic) treatment of cell e.s.d.'s is used for estimating e.s.d.'s involving l.s. planes.
Refinement. Refinement of *F*^2^ against ALL reflections. The weighted *R*-factor *wR* and goodness of fit *S* are based on *F*^2^, conventional *R*-factors *R* are based on *F*, with *F* set to zero for negative *F*^2^. The threshold expression of *F*^2^ > σ(*F*^2^) is used only for calculating *R*-factors(gt) *etc*. and is not relevant to the choice of reflections for refinement. *R*-factors based on *F*^2^ are statistically about twice as large as those based on *F*, and *R*- factors based on ALL data will be even larger.

### Fractional atomic coordinates and isotropic or equivalent isotropic displacement parameters (Å^2^)

**Table:**

	*x*	*y*	*z*	*U*_iso_*/*U*_eq_	
C1	0.66295 (19)	0.0759 (2)	0.21188 (13)	0.0223 (4)	
H1	0.6598	0.1397	0.2563	0.027*	
C2	0.77558 (18)	0.0545 (3)	0.18405 (13)	0.0235 (4)	
H2	0.8465	0.1018	0.2102	0.028*	
C3	0.78166 (17)	−0.0388 (2)	0.11638 (12)	0.0193 (4)	
C4	0.90160 (18)	−0.0652 (3)	0.08374 (15)	0.0266 (4)	
H4A	0.9514	−0.1379	0.1175	0.032*	
H4B	0.945	0.0319	0.0826	0.032*	
H4C	0.8849	−0.1066	0.0303	0.032*	
C5	0.67277 (17)	−0.1087 (2)	0.08084 (12)	0.0180 (4)	
H5	0.6732	−0.1716	0.0358	0.022*	
C6	0.56307 (16)	−0.0844 (2)	0.11273 (11)	0.0154 (3)	
C7	0.44364 (16)	−0.1594 (2)	0.07909 (11)	0.0157 (3)	
C8	0.43269 (17)	−0.2639 (2)	0.01571 (11)	0.0178 (4)	
H8	0.5013	−0.2879	−0.0092	0.021*	
C9	0.31873 (18)	−0.3335 (2)	−0.01087 (12)	0.0188 (4)	
C10	0.3034 (2)	−0.4469 (3)	−0.07953 (14)	0.0275 (4)	
H10C	0.2531	−0.5329	−0.0668	0.033*	
H10B	0.383	−0.4849	−0.0886	0.033*	
H10A	0.2643	−0.3956	−0.1271	0.033*	
C11	0.21939 (18)	−0.2944 (3)	0.02924 (12)	0.0220 (4)	
H11	0.1423	−0.3396	0.0142	0.026*	
C12	0.23656 (17)	−0.1886 (3)	0.09121 (12)	0.0227 (4)	
H12	0.1693	−0.163	0.1171	0.027*	
N1	0.55839 (15)	0.0081 (2)	0.17723 (10)	0.0179 (3)	
N2	0.34579 (14)	−0.1203 (2)	0.11628 (10)	0.0180 (3)	
N3	0.39927 (16)	0.3475 (2)	0.16842 (11)	0.0222 (3)	
N4	0.44300 (15)	−0.0611 (2)	0.36776 (10)	0.0202 (3)	
O1	0.41779 (13)	0.27826 (17)	0.23681 (8)	0.0216 (3)	
O2	0.36572 (15)	0.2656 (2)	0.10800 (9)	0.0288 (3)	
O3	0.41477 (16)	0.4902 (2)	0.16496 (11)	0.0316 (4)	
O4	0.37061 (13)	−0.10009 (18)	0.30217 (9)	0.0224 (3)	
O5	0.50246 (15)	0.06073 (19)	0.36678 (10)	0.0287 (3)	
O6	0.44574 (16)	−0.1479 (2)	0.42578 (10)	0.0360 (4)	
O7	0.19144 (14)	0.0920 (2)	0.20178 (10)	0.0242 (3)	
H7A	0.169 (3)	0.185 (4)	0.1905 (18)	0.036 (8)*	
H7B	0.156 (4)	0.067 (4)	0.243 (2)	0.058 (11)*	
Zn1	0.37859 (2)	0.04435 (3)	0.207009 (13)	0.01710 (7)	

### Atomic displacement parameters (Å^2^)

**Table:**

	*U*^11^	*U*^22^	*U*^33^	*U*^12^	*U*^13^	*U*^23^
C1	0.0188 (9)	0.0263 (11)	0.0217 (10)	−0.0027 (7)	0.0022 (7)	−0.0054 (8)
C2	0.0149 (8)	0.0291 (11)	0.0259 (10)	−0.0042 (8)	0.0002 (7)	−0.0003 (8)
C3	0.0117 (8)	0.0217 (9)	0.0246 (9)	0.0021 (7)	0.0031 (7)	0.0059 (8)
C4	0.0132 (8)	0.0314 (12)	0.0360 (12)	0.0009 (8)	0.0064 (8)	0.0022 (9)
C5	0.0152 (8)	0.0191 (9)	0.0203 (9)	0.0015 (7)	0.0046 (7)	0.0011 (7)
C6	0.0133 (8)	0.0175 (9)	0.0157 (8)	0.0017 (6)	0.0025 (6)	0.0032 (6)
C7	0.0106 (8)	0.0210 (9)	0.0158 (8)	−0.0002 (6)	0.0024 (6)	0.0025 (7)
C8	0.0152 (8)	0.0207 (9)	0.0182 (9)	0.0006 (7)	0.0047 (6)	0.0011 (7)
C9	0.0185 (9)	0.0212 (10)	0.0164 (9)	−0.0008 (7)	0.0009 (7)	0.0019 (7)
C10	0.0268 (10)	0.0263 (11)	0.0283 (11)	−0.0032 (9)	0.0003 (8)	−0.0058 (9)
C11	0.0142 (8)	0.0295 (11)	0.0215 (9)	−0.0022 (8)	−0.0006 (7)	0.0022 (8)
C12	0.0106 (8)	0.0368 (12)	0.0209 (9)	−0.0003 (8)	0.0026 (7)	0.0007 (8)
N1	0.0139 (7)	0.0210 (8)	0.0189 (8)	0.0010 (6)	0.0026 (6)	−0.0005 (6)
N2	0.0112 (7)	0.0250 (9)	0.0178 (8)	0.0015 (6)	0.0024 (5)	−0.0008 (6)
N3	0.0190 (8)	0.0264 (9)	0.0232 (9)	0.0038 (7)	0.0099 (6)	0.0031 (7)
N4	0.0160 (7)	0.0248 (9)	0.0189 (8)	0.0005 (6)	−0.0006 (6)	−0.0003 (6)
O1	0.0255 (7)	0.0218 (7)	0.0182 (7)	0.0021 (6)	0.0055 (5)	0.0024 (5)
O2	0.0269 (8)	0.0409 (10)	0.0195 (7)	0.0022 (7)	0.0063 (6)	−0.0033 (6)
O3	0.0360 (9)	0.0257 (8)	0.0370 (9)	0.0029 (7)	0.0185 (7)	0.0095 (7)
O4	0.0209 (7)	0.0262 (7)	0.0185 (7)	−0.0056 (6)	−0.0029 (5)	0.0013 (6)
O5	0.0281 (8)	0.0273 (8)	0.0299 (8)	−0.0102 (6)	0.0011 (6)	−0.0023 (6)
O6	0.0388 (9)	0.0395 (10)	0.0257 (8)	−0.0091 (8)	−0.0103 (7)	0.0133 (7)
O7	0.0182 (7)	0.0229 (8)	0.0333 (9)	0.0056 (6)	0.0102 (6)	0.0052 (6)
Zn1	0.01435 (11)	0.02035 (12)	0.01732 (12)	0.00208 (9)	0.00476 (7)	−0.00014 (9)

### Geometric parameters (Å, °)

**Table:**

C1—N1	1.342 (3)	C10—H10B	0.96
C1—C2	1.387 (3)	C10—H10A	0.96
C1—H1	0.93	C11—C12	1.374 (3)
C2—C3	1.399 (3)	C11—H11	0.93
C2—H2	0.93	C12—N2	1.344 (2)
C3—C5	1.392 (3)	C12—H12	0.93
C3—C4	1.506 (3)	Zn1—N1	2.1157 (16)
C4—H4A	0.96	Zn1—N2	2.0727 (17)
C4—H4B	0.96	N3—O3	1.238 (3)
C4—H4C	0.96	N3—O2	1.248 (2)
C5—C6	1.394 (3)	N3—O1	1.285 (2)
C5—H5	0.93	N4—O6	1.223 (2)
C6—N1	1.350 (3)	N4—O5	1.231 (2)
C6—C7	1.494 (2)	N4—O4	1.309 (2)
C7—N2	1.352 (2)	Zn1—O1	2.0965 (15)
C7—C8	1.385 (3)	Zn1—O2	2.5143 (16)
C8—C9	1.397 (3)	Zn1—O4	2.0340 (15)
C8—H8	0.93	Zn1—O7	2.0752 (15)
C9—C11	1.395 (3)	O7—H7A	0.84 (3)
C9—C10	1.501 (3)	O7—H7B	0.87 (4)
C10—H10C	0.96		
			
N1—C1—C2	122.57 (19)	H10B—C10—H10A	109.5
N1—C1—H1	118.7	C12—C11—C9	119.35 (18)
C2—C1—H1	118.7	C12—C11—H11	120.3
C1—C2—C3	119.52 (18)	C9—C11—H11	120.3
C1—C2—H2	120.2	N2—C12—C11	123.24 (18)
C3—C2—H2	120.2	N2—C12—H12	118.4
C5—C3—C2	117.51 (17)	C11—C12—H12	118.4
C5—C3—C4	120.95 (19)	C1—N1—C6	118.65 (17)
C2—C3—C4	121.53 (18)	C1—N1—Zn1	126.65 (14)
C3—C4—H4A	109.5	C6—N1—Zn1	114.51 (12)
C3—C4—H4B	109.5	C12—N2—C7	118.13 (18)
H4A—C4—H4B	109.5	C12—N2—Zn1	125.71 (14)
C3—C4—H4C	109.5	C7—N2—Zn1	116.15 (13)
H4A—C4—H4C	109.5	O3—N3—O2	123.01 (19)
H4B—C4—H4C	109.5	O3—N3—O1	119.53 (18)
C3—C5—C6	120.01 (18)	O2—N3—O1	117.46 (18)
C3—C5—H5	120	O6—N4—O5	124.81 (17)
C6—C5—H5	120	O6—N4—O4	117.51 (17)
N1—C6—C5	121.72 (17)	O5—N4—O4	117.68 (17)
N1—C6—C7	115.43 (16)	N3—O1—Zn1	103.06 (12)
C5—C6—C7	122.85 (18)	N4—O4—Zn1	114.88 (12)
N2—C7—C8	121.69 (17)	Zn1—O7—H7A	116 (2)
N2—C7—C6	115.03 (17)	Zn1—O7—H7B	118 (2)
C8—C7—C6	123.26 (17)	H7A—O7—H7B	105 (3)
C7—C8—C9	120.09 (17)	O4—Zn1—N2	98.18 (7)
C7—C8—H8	120	O4—Zn1—O7	90.28 (6)
C9—C8—H8	120	N2—Zn1—O7	91.88 (6)
C11—C9—C8	117.48 (18)	O4—Zn1—O1	115.06 (6)
C11—C9—C10	120.98 (18)	N2—Zn1—O1	146.72 (6)
C8—C9—C10	121.54 (18)	O7—Zn1—O1	89.42 (6)
C9—C10—H10C	109.5	O4—Zn1—N1	103.76 (6)
C9—C10—H10B	109.5	N2—Zn1—N1	78.38 (6)
H10C—C10—H10B	109.5	O7—Zn1—N1	163.82 (7)
C9—C10—H10A	109.5	O1—Zn1—N1	91.77 (6)
H10C—C10—H10A	109.5		
			
N1—C1—C2—C3	1.2 (3)	C6—C7—N2—Zn1	3.6 (2)
C1—C2—C3—C5	−1.2 (3)	O3—N3—O1—Zn1	−179.98 (15)
C1—C2—C3—C4	179.8 (2)	O2—N3—O1—Zn1	0.18 (19)
C2—C3—C5—C6	0.1 (3)	O6—N4—O4—Zn1	−176.91 (15)
C4—C3—C5—C6	179.08 (19)	O5—N4—O4—Zn1	3.7 (2)
C3—C5—C6—N1	1.1 (3)	N4—O4—Zn1—N2	147.48 (13)
C3—C5—C6—C7	−178.21 (18)	N4—O4—Zn1—O7	−120.58 (14)
N1—C6—C7—N2	2.1 (2)	N4—O4—Zn1—O1	−31.09 (15)
C5—C6—C7—N2	−178.61 (18)	N4—O4—Zn1—N1	67.52 (14)
N1—C6—C7—C8	−176.59 (18)	C12—N2—Zn1—O4	73.10 (18)
C5—C6—C7—C8	2.7 (3)	C7—N2—Zn1—O4	−107.81 (14)
N2—C7—C8—C9	−0.7 (3)	C12—N2—Zn1—O7	−17.46 (18)
C6—C7—C8—C9	177.87 (18)	C7—N2—Zn1—O7	161.64 (15)
C7—C8—C9—C11	−0.7 (3)	C12—N2—Zn1—O1	−109.26 (19)
C7—C8—C9—C10	179.82 (19)	C7—N2—Zn1—O1	69.83 (18)
C8—C9—C11—C12	1.3 (3)	C12—N2—Zn1—N1	175.56 (18)
C10—C9—C11—C12	−179.2 (2)	C7—N2—Zn1—N1	−5.34 (14)
C9—C11—C12—N2	−0.6 (3)	N3—O1—Zn1—O4	−170.58 (10)
C2—C1—N1—C6	0.0 (3)	N3—O1—Zn1—N2	12.00 (17)
C2—C1—N1—Zn1	−174.67 (16)	N3—O1—Zn1—O7	−80.55 (12)
C5—C6—N1—C1	−1.1 (3)	N3—O1—Zn1—N1	83.32 (12)
C7—C6—N1—C1	178.23 (17)	C1—N1—Zn1—O4	−83.06 (18)
C5—C6—N1—Zn1	174.17 (14)	C6—N1—Zn1—O4	102.12 (14)
C7—C6—N1—Zn1	−6.5 (2)	C1—N1—Zn1—N2	−178.76 (19)
C11—C12—N2—C7	−0.8 (3)	C6—N1—Zn1—N2	6.43 (14)
C11—C12—N2—Zn1	178.30 (16)	C1—N1—Zn1—O7	127.3 (2)
C8—C7—N2—C12	1.4 (3)	C6—N1—Zn1—O7	−47.5 (3)
C6—C7—N2—C12	−177.27 (17)	C1—N1—Zn1—O1	33.29 (18)
C8—C7—N2—Zn1	−177.75 (14)	C6—N1—Zn1—O1	−141.52 (14)

### Hydrogen-bond geometry (Å, °)

**Table:**

*D*—H···*A*	*D*—H	H···*A*	*D*···*A*	*D*—H···*A*
O7—H7A···O4^i^	0.85 (3)	1.90 (3)	2.724 (2)	163 (3)
O7—H7B···O3^ii^	0.87 (4)	1.94 (4)	2.799 (2)	174 (3)
C5—H5···O2^iii^	0.93	2.53	3.420 (2)	161
C8—H8···O2^iii^	0.93	2.36	3.230 (2)	156
C11—H11···O6^iv^	0.93	2.44	3.284 (3)	151

Symmetry codes: (i) −*x*+1/2, *y*+1/2, −*z*+1/2; (ii) −*x*+1/2, *y*−1/2, −*z*+1/2; (iii) −*x*+1, −*y*, −*z*; (iv) *x*−1/2, −*y*−1/2, *z*−1/2.
